# Metformin Is Associated With Slightly Reduced Risk of Colorectal Cancer and Moderate Survival Benefits in Diabetes Mellitus: A Meta-Analysis

**DOI:** 10.1097/MD.0000000000002749

**Published:** 2016-02-18

**Authors:** Xing-kang He, Ting-ting Su, Jian-min Si, Lei-min Sun

**Affiliations:** From the Department of Gastroenterology, Sir Run Run Shaw Hospital, Zhejiang University Medical School (X-KH, T-TS, J-MS, L-MS); and Institute of Gastroenterology, Zhejiang University (IGZJU), Hangzhou, P. R. China (X-KH, T-TS, J-MS, L-MS).

## Abstract

To systematically assess the effect of metformin on colorectal cancer (CRC) risk and mortality in type 2 diabetes mellitus (T2DM) patients.

We conducted a systematic search of PubMed, Web of Science, and the Cochrane Library databases for relevant articles before August 2015. Two investigators identified and extracted data independently. We adopted adjusted estimates to calculate summary estimates with 95% confidence interval (CI) using either a fixed-effects or a random-effects model. Subgroup and sensitivity analyses were conducted to evaluate the robustness of the pooled results. The risk of publication bias was assessed by examining funnel plot asymmetry as well as Begg test and Egger test.

Fifteen studies on CRC incidence and 6 studies on CRC survival were finally included in our meta-analysis. The pooled odds ratio (OR) of observational studies illustrated that a slight 10% reduction of CRC incidence was associated with metformin use (OR = 0.90, 95% CI: 0.85–0.96). Furthermore, the pooled hazard ratio (HR) revealed an improved survival outcome for metformin users in CRC patients compared to nonusers (HR = 0.68, 95% CI: 0.58–081). There was no publication bias across studies.

Our meta-analysis demonstrated that metformin therapy could slightly reduce CRC incidence and moderately improve the survival outcomes in patients with T2DM. More prospective studies are warranted to certify this protective association.

## INTRODUCTION

Colorectal cancer (CRC) is the second most commonly prevalent cancer in males and the third most commonly malignant disease in females in America.^1^ It is a leading cause of cancer-related deaths in America^2^, Europe^3^, and Asia.^4^ Regular screening with colonoscopy in high-risk population is a preferred approach recommended by the American Cancer Society (ACS).^5^ Given limitations of screening examinations, unfortunately, there is a great interest on exploring chemopreventive drugs to reduce the huge burden of CRC.

Metformin, as a first-line treatment for type 2 diabetes mellitus (T2DM), is reported reducing the incidence of many cancers, including CRC.^6,7^ Previous studies suggested that T2DM is closely related with the risk and prognosis of CRC,^8–10^ since they share several common risk factors, such as obesity, smoking, drinking, the western diet, and lack of exercise.^11^ T2DM may contribute to the development of CRC through several mechanisms, including hyperglycemia, oxidative stress, and chronic inflammation.^9^ Encouragingly, a serials of epidemiologic studies,^12–14^ but not all,^15,16^ had shown a lower risk and mortality of CRC associated with metformin use. Several basic researches also demonstrated that metformin inhibited cancer cell proliferation, metabolism, and angiogenesis through activation of adenosine monophosphate-activated protein kinase (AMPK) and inhibition of mammalian target of rapamycin (mTOR) signaling pathway.^17–19^ Metformin may have multiple activities against tumor, which represent a promising perspective in cancer therapy.^20^ To date, though the antineoplastic effects of metformin are biologically plausible, existing data remain controversial. For example, several studies^15,16^ have shown that metformin does not reduce the incidence of CRC in patients with T2DM.

Considering these controversial contexts, we performed a meta-analysis based on existing observational studies and randomized controlled trials (RCTs) to determine whether use of metformin may protect T2DM patients against CRC. Since high prevalence and poor prognosis of CRC, a potential antitumor role of metformin would markedly impact on clinical and public health.

## METHODS

This meta-analysis was conducted according to the Preferred Reporting Items for Systematic reviews and Meta-Analysis (PRISMA) guidelines.^21^

### Search Strategy

We (HXK and STT) independently searched Medline, Web of Science, and the Cochrane Library databases for all relevant studies before August 2015. Medical subject heading (Mesh) terms and keywords were used in the search included “metformin,” “biguanide,” “colon neoplasm,” and “colorectal cancer.” Two authors reviewed the titles and abstracts of studies identified in the search independently in order to exclude unrelated studies. We examined the remaining full articles and references to determine whether it contained any additional papers.

### Eligibility Criteria

Eligible articles were considered in this meta-analysis if they met the following criteria: original articles reported estimated risks with 95% confidence interval (CI); evaluated association between CRC and metformin use; T2DM was identified before CRC diagnosis based on medical or pathological diagnosis; studies published in English were included. When there were multiple publications from the same cohort, we extracted information from the most recent comprehensive study.

### Data Extraction and Quality Assessment

Two researchers (HXK and STT) extracted data from included studies independently by scrutinizing the full text. The following information were collected from eligible articles: authors, year, design, location, time period, exposure ascertainment, outcome assessment, total subjects, colon cancer cases and confounding variables adjusted, and so on. In order to better understand the risk of bias among included studies, the Newcastle-Ottawa Scale^22^ was applied for quality assessment in observational studies. The Cochrane Collaboration's tool was also used to assess the risk of bias in RCTs. All methodological quality of eligible studies were performed by 2 authors independently (HXK and STT). Any discrepancies were resolved by discussions or with the third researcher (SLM).

### Statistical Analysis

Pooled ORs (HRs) and 95% CI were calculated using a random-effects model^23^ if the heterogeneity was considerable, and a fixed-effects model was performed otherwise. Adjusted estimates reported in studies were used for meta-analysis in order to account for confounding factors. We assessed heterogeneity among individual studies by 2 methods: Cochran Q test and *I*^2^.^24^ Statistically significant for heterogeneity was considered if *P* ≤ 0.05 and/or *I*^2^ > 30%. In order to investigate sources of heterogeneity, we performed subgroup analyses^25^ by grouping study location, design, adjusted for other antidiabetic medications (ADMs). Besides we conducted sensitivity analyses by excluding 1 study each time and rerunning the analysis to verify the robustness of the overall results. Publication bias was assessed by conducting statistical tests for funnel plot asymmetry as well as Egger test and Begg test. A probability level <0.05 was considered statistically significant and all *P* values were 2 tailed. All statistical analyses were conducted using Stata software (version 11.0; StataCorp, College Station, TX).

## RESULTS

**FIGURE 1:**
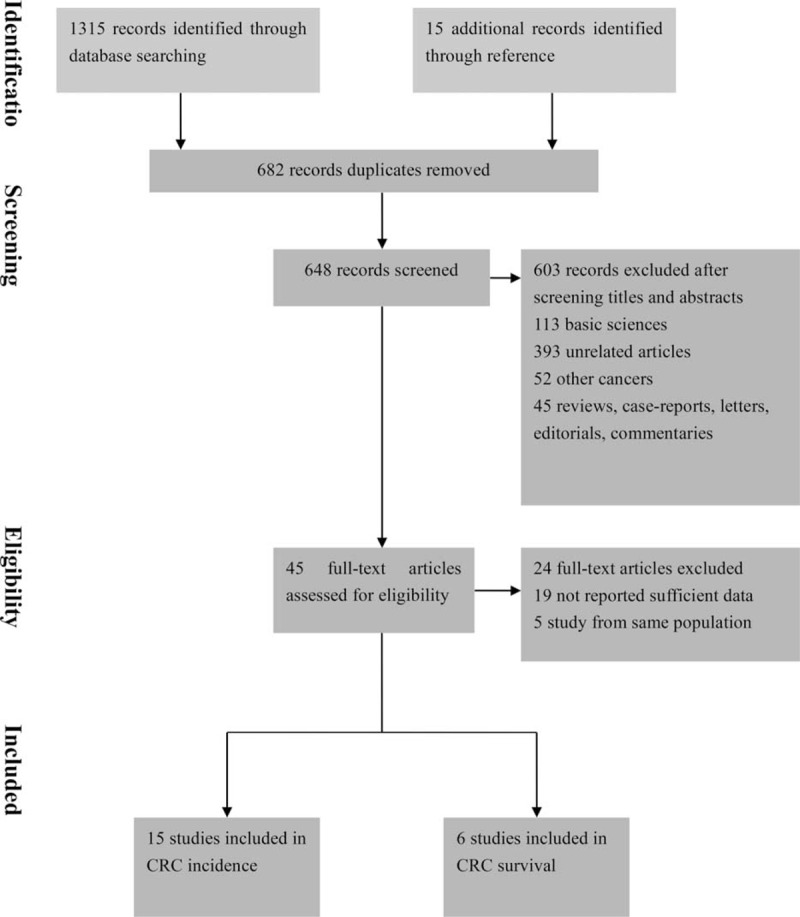
Flow diagram summarizing study identification and selection.

There were 1330 studies that were identified by the search strategy. Among them, only 20 observation studies and one RCT were finally included in this meta-analysis (Figure 1). These studies cumulatively included 16,786 cases of CRC in 1,086,268 patients with T2DM. There were three Taiwanese studies^26–28^ from the same cohort, therefore, only one^27^ of them was included in the analysis for metformin and CRC incidence. Likewise, four United Kingdom (UK) studies^15,29–31^ from the same cohort and only one^30^ of them was included.

### Characteristics and Quality of Included Studies

The characteristics of included studies are shown in Tables 1 and 2. Fifteen studies of them evaluated the association between CRC incidence and metformin, while other 6 studies assessed survival benefits associated with metformin exposure. Seventeen studies were from the Western population (7 based in the United States (US), 10 based in Europe), 3 studies were performed in the Asian population, and 1 was a multicenter RCT across the US, Europe, and Asia. Seventeen selected studies were published in recent 5 years (2010–2015). Two articles^16,32^ reported 4 different cohorts, while an article^33^ reported colon and rectal cancer, respectively. In most studies, exposure was ascertained from pharmacy database and outcome assessment was based on standard diagnostic codes. Nonresponse rate in case–control studies and duration of follow-up in cohort were inconsistently reported.

**TABLE 1:**
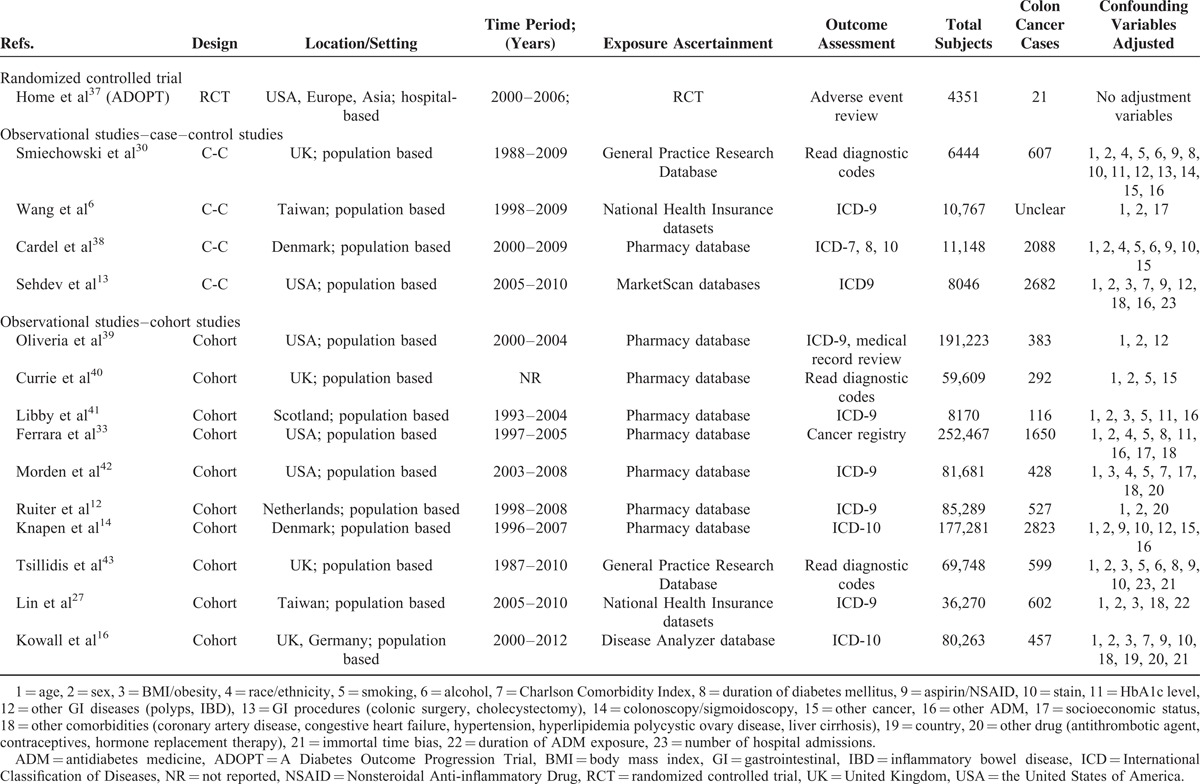
Characteristics of Included Studies Assessing the Risk of Colorectal Cancer in Patients With Diabetes Mellitus Treated With Metformin

**TABLE 2:**
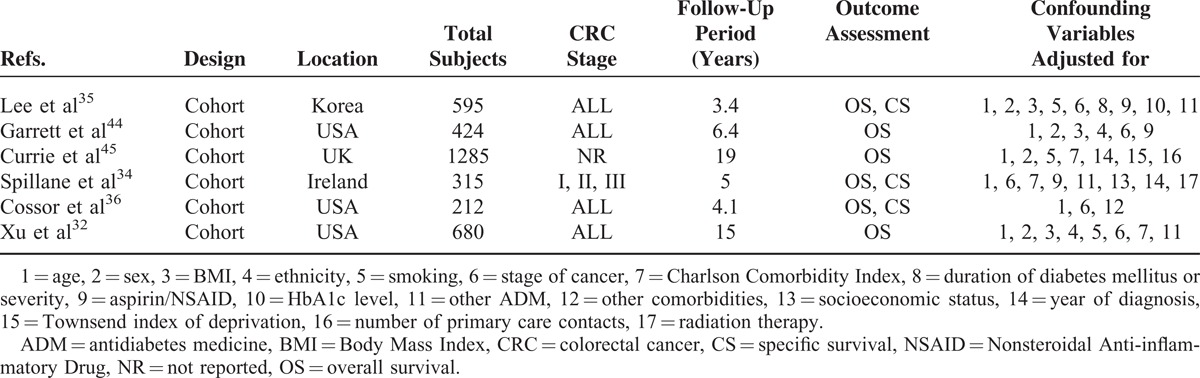
Characteristics of Included Studies Assessing the Prognosis of Colorectal Cancer in Patients With Diabetes Mellitus Treated With Metformin

The overall methodological quality was moderate to high (Tables 3–5). Using the Newcastle-Ottawa scale quality tool, the quality of included studies was moderate or high. The quality of the randomized trials was moderate according to the Cochrane Collaboration's tool.

**TABLE 3:**
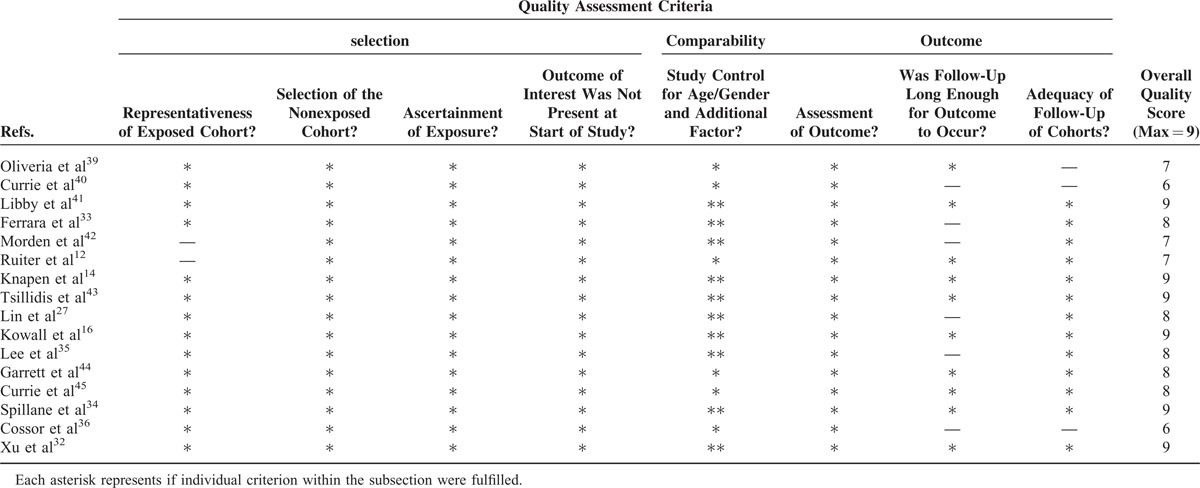
Newcastle-Ottawa Scale for Assessment of Quality of in Included Cohort Studies

**TABLE 4:**
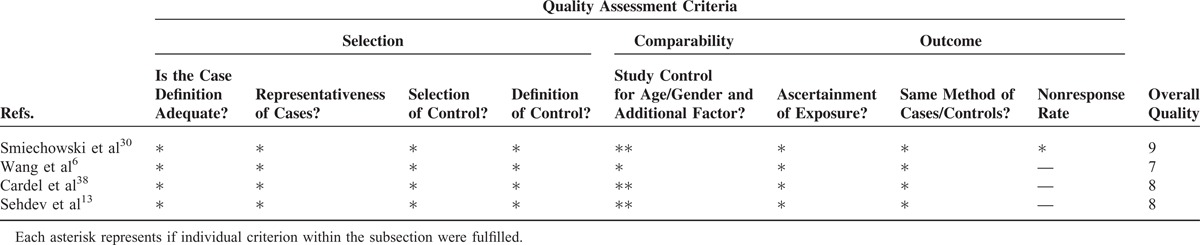
Newcastle-Ottawa Scale for Assessment of Quality of Included Case–Control Studies

**TABLE 5:**
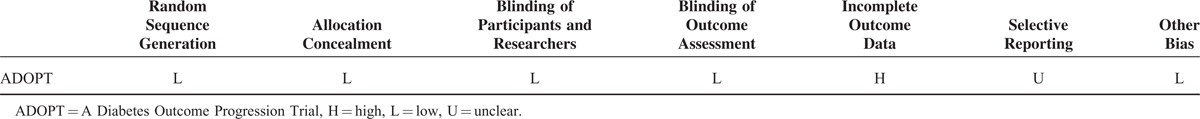
Cochrane Collaboration's Tool for Assessment of Quality of Randomized Controlled Trials

### Metformin Exposure and Risk of CRC

**FIGURE 2:**
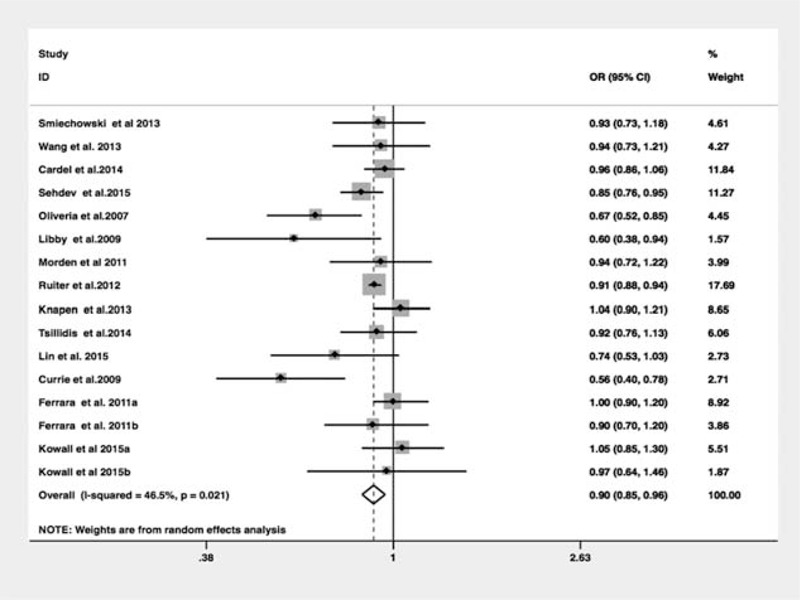
Meta-analysis of the association between metformin use and colorectal cancer risk in patients with diabetes mellitus.

**FIGURE 3:**
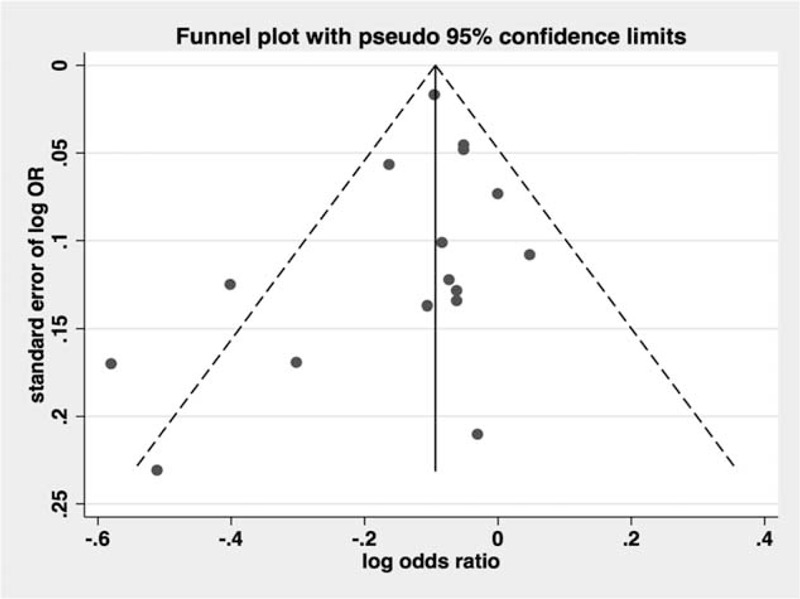
Funnel plot analysis to detect publication bias.

Among the 14 observational studies that reported CRC incidence, 5 demonstrated an apparent protective association and the other 9 studies showed no statistically significant relationship. The pooled analyses of observational studies demonstrated that the use of metformin was associated with a statistically significant 10% reduction in CRC incidence among T2DM patients (OR = 0.90, 95% CI: 0.85–0.96) (Figure 2), which was consistent with previous meta-analysis. The results of subgroup analyses for the association between metformin use and CRC risk are demonstrated in Table 6. Importantly, we performed sensitivity analyses by excluding 1 article each time and recalculated the pooled OR for remaining studies. Results demonstrated overall pooled estimates were robust and the chemopreventive effect of metformin persisted in CRC patients with T2DM. There was considerable heterogeneity among studies (*I*^2^ = 46.5%, *P* = 0.02), which could be partly due to study design. There was no evidence of publication bias in our analysis, based on the Egger test (*P* = 0.27) or Begg test (P = 0.14), and on visual inspection of the funnel plot (Figure 3).

**TABLE 6:**
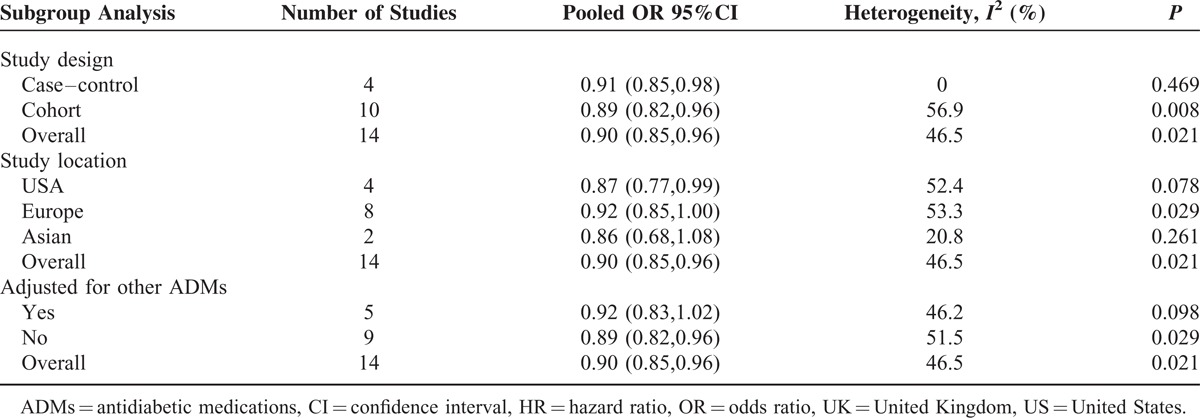
Subgroup Analysis of Studies Comparing the Association Between CRC Risk and Metformin

### Metformin and Morality of CRC

**FIGURE 4:**
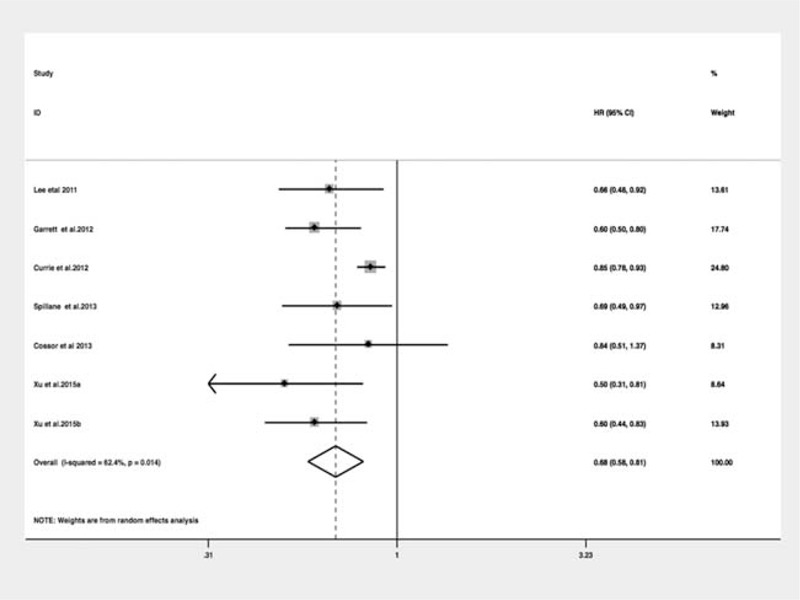
Meta-analysis of the association between metformin use and all-cause mortality in patients with diabetes mellitus.

Among the six selected studies, all reported overall survival (OS) and three^34–36^ also presented CRC-specific survival (CS). The pooled HR of OS was 0.68 (95% CI: 0.58–081) (Figure 4), with some evidence of heterogeneity (*I*^2^ = 62.4%, *P* = 0.01). The pooled HR of CS was 0.66 (95% CI: 0.50–0.87), with no evidence of heterogeneity (*I*^2^ = 0%, *P* = 0.88). Our study showed that metformin use in CRC patients with T2DM moderately reduced both all-cause death and CRC-specific mortality. Subgroup and sensitivity analyses were not performed since the number of included studies was limited. Substantial heterogeneity was present among OS and no heterogeneity existed for CS. Because of the limited number of included studies, it was difficult to confirm whether the publication bias exists in our meta-analysis.^46^

## DISCUSSION

Based on 20 observational studies and 1 RCT, our meta-analysis showed metformin was associated with a slight, yet statistically significant, protective effect (10% risk reduction) on CRC risk among patients with T2DM. The benefits associated with metformin were stable even after sensitivity analyses. It was also in line with results of previous meta-analysis.^47,48^ Though identified in observational studies, the potential antineoplastic effect of metformin is unproven in the RCT (OR = 0.69, 95% CI: 0.26–1.82). This may be due to the fact that the trial was not primarily designed to explore the effect of metformin on CRC risk, which inevitably introduced some bias into the trial. Besides, 1 RCT might have no enough power to detect a significant association between metformin with CRC risk. Therefore, further specially designed RCTs are needed to confirm this protective effect.

Notably current studies indicated that the magnitude of chemopreventive effect was not as obvious as previous studies.^48^ Recently, more researchers were aware of shortcoming and potential bias of observational studies.^49,50^ In order to avoid overestimation the effect of metformin, authors minimized time-related bias^49,50^ and adjusted more confounding factors as far as possible. Subgroup analyses also suggested that the protective association between metformin and CRC risk was not different among different regions (US OR = 0.87, 95% CI: 0.77–0.99; Europe OR = 0.92, 95% CI: 0.85–1.00; Asian OR = 0.86, 95% CI: 0.68–1.08). More importantly, survival advantages were observed among CRC patients with T2DM in our analysis. Patients taking metformin had a better prognosis compared with nonusers, which achieved estimated OS benefits of 32%.

Although previous studies indicated that metformin was associated with a reduction in CRC risk, potential biologic mechanisms underlying the antitumor effect of metformin was still pending. There is a growing body of evidence indicating that metformin exerts the anticancer activity through its systemic effects as well as cellular effects. The systemic effects of metformin can potentially counteract the Warburg effect by reducing hyperglycemia.^51^ Warburg effect is a crucial metabolic feature in cancer cells that facilitates bypass senescence.^52^ The cellular effects are associated with activation of AMPK and consequently inhibition of mTOR pathway,^17,53^ which plays a critical role in cell proliferation and carcinogenesis among many tumors. Activation of mTOR closely correlates with cancer progression, resistance to chemotherapy, and poor prognosis.^54^ Furthermore, metformin may also promote tumor cell senescence through suppressing cyclin D1 expression.^55^ The antitumor effects have also been illustrated in animal models of CRC. Tomimoto et al^18^ reported that metformin could suppress intestinal polyposis in the adenomatous polyposis coli (APC^Min/+^) mice. Besides, metformin could also inhibit the formation of colorectal aberrant crypt foci in the murine model of azoxymethane-induced colitis-associated cancer.^56^ These evidence from in vivo and in vitro strengthen the role of metformin as one of the promising candidates for cancer therapeutics.

The strength of our systematic analysis consists in including comprehensive studies, large numbers of patients, as well as assessment of the survival benefits between metformin and CRC. Zhang et al^48^ firstly performed a meta-analysis of metformin and CRC risk in 2011, however, they included only 4 studies and did not perform subgroup or sensitivity analysis since limited numbers. Recently, Singh et al^47^ performed a meta-analysis of ADMs and CRC risk. They included 10 articles and failed to evaluate specifically metformin and CRC. Both studies did not assess the effect of metformin on CRC survival. In fact, both of them showed a chemopreventive effect of metformin, though variable in magnitude. The magnitude of protective effect in our study was less evident comparing with the meta-analysis in 2011 and similar with that of Singh's analysis. This may be due to the larger numbers of the included studies. Meanwhile, we speculated that recently published studies avoided time-related bias and took a wide range of confounding variables into consideration. The conclusion of our study about metformin affection on CRC risk might be more scientific and credible. CRC survival benefits associated with metformin in our results also supported the antitumor effect of metformin. Of note, adjusted estimates were used to calculate the summary results instead of unadjusted ones in order to avoid potential confounding factors. Besides we performed subgroup and sensibility analyses to ensure stability of the association and identify factors responsible for heterogeneity.

However, our study also has several limitations that merit further consideration. Firstly, our pooled results were based on data from observational studies, while only 1 RCT is feasible. Observational studies had methodical shortcomings and are prone to time-related biases, such as immortal time bias and time-lagging issues.^49^ This may potentially overestimate the apparently protective effect of metformin. Secondly, the adjusted potential covariates of included studies were incomplete and inconsistent. Moreover, some confounding factors such as dietary consumptions, physical activity, and screening colonoscopy were not well adjusted for included studies. Thirdly, the included studies were limited in reporting dose and duration of metformin use among CRC patients with T2DM. Hence, neither dose–response or duration–response association between metformin use and risk of CRC could be established. Finally, this meta-analysis was restricted to English language studies, which might introduce publication bias.

In summary, our meta-analysis demonstrated metformin use might be associated with a lower risk and better prognosis of CRC in diabetic patients based on current evidence. These data highlight the role of metformin as a potential candidate for chemopreventive drugs on CRC patients with T2DM. However, further investigations, especially well-designed RCTs, are expected to substantiate these benefits from early observational studies.
